# Magnitude and predictors of poor glycemic control among patients with diabetes attending public hospitals of Western Ethiopia

**DOI:** 10.1371/journal.pone.0247634

**Published:** 2021-02-25

**Authors:** Adugna Oluma, Muktar Abadiga, Getu Mosisa, Werku Etafa

**Affiliations:** 1 Department of Nursing, School of Nursing, and Midwifery, Institute of Health Sciences, Wollega University, Nekemte, Ethiopia; 2 Department of Pediatrics and Neonatal Nursing, School of Nursing and Midwifery, Institute of Health Sciences, Wollega University, Nekemte, Ethiopia; West Virginia University, UNITED STATES

## Abstract

**Background:**

Diabetes is one of the most prevalent non-communicable diseases globally, which rapidly is increasing in developing countries. Ethiopia is also facing growing morbidity and mortality related to diabetes complications. Thus, dealing with glycemic control is essential for controlling the development of devastating acute and chronic complications related to diabetes. Therefore, this study aims to assess the magnitude and predictors of poor glycemic control among diabetic patients in western Ethiopia.

**Methods:**

The cross-sectional study design was employed on a sample of 423 diabetic patients. A systematic random sampling method was employed. An interviewer-administered structured questionnaire was used. The data entered into Epi data version 3.1 and exported into Statistical Package for the Social Sciences window version 24 for analysis. All variables significant at p-<0.25 in bivariate were entered into multivariate analysis. The multivariable logistic regressions were used to determine predictors’ poor glycemic control by considering the Adjusted Odds Ratio at CI 95% and the significance level was set at p <0.05.

**Results:**

The magnitude of poor glycemic control was 64.1%. Being females (AOR = 1.684,95%CI = 1.066,2.662), duration of diabetes >8years (AOR = 2.552,95%CI = 1.397, 4.665), presence of diabetes complication (AOR = 2.806,95%CI = 1.594,4.941), negligence of blood glucose test at home (AOR = 1.720, 95%CI = 1.078, 2.743), poor self-care behavior (AOR = 1.787, 95%CI = 1.083,2.959) and poor self-efficacy (AOR = 1.934, 95%CI = 1.078,3.469) were significant predictors of poor glycemic control.

**Conclusion:**

The proportion of poor glycemic control was high which was nearly comparable to that reported from many countries. This could be due to factors that were significantly associated with poor glycemic control like lack of home blood glucose test, increased duration of diabetes, presence of diabetes complications, poor self-efficacy, and poor self-care behaviors. Each were significant independent predictors of poor glycemic control. Thus, we recommend patients with diabetes and health care providers enhancing self-monitoring practices, and preventing potential complications should be a priority concern to improve blood glucose levels. Further studies are also recommended to explore important factors which were not identified by the current study.

## Introduction

Diabetes mellitus (DM) is a chronic disease caused by inherited and/or acquired deficiency in production and/ or decreased tissue sensitivity to insulin action [1]. Globally, an estimated 463 million adults (20–79 years) are living with diabetes; by 2045, these numbers rise to 700 million [2]. Approximately, about 79% of adults with diabetes were living in low and middle-income countries. In Ethiopia, the prevalence was dramatically increasing from 3.8% - 5.2% [3].

Diabetes mellitus is a serious global public health problem that affects the whole life of the people in terms of their biological, psychological, and social effects. The cost of paying for diabetes is rising dramatically due to complications ranging from an increased risk of heart attacks, strokes, and amputations to blindness and kidney damage [4, 5].

The study showed that complications and death from diabetes occur from poorly controlled blood glucose levels. An intensive blood glucose control is a cornerstone to decrease complications related to chronic diseases including diabetes [6]. A basic way of monitoring glycemic levels is by using Glycated hemoglobin (HgA1c). Glycated hemoglobin is an indicator recommended for monitoring appropriately glycemic control status. It indicates the amount of blood glucose within the last 3 months [7].

Most kinds of literature deal with the correlation between type II diabetes mellitus with poor glycemic control [8, 9]. However, the prevalence of type I diabetes mellitus was also dramatically increasing in younger individuals. Therefore, dealing with the determinants of poor glycemic control in both types I and type II diabetes is pivotal to control both micro vascular and macro vascular diabetic complications that endanger the health of the whole public [10].

The basic strategies for effective management of diabetes are educating the public that linked behavioral changes. This treatment plan includes adequate glycemic control through self-monitoring of blood glucose levels and measurement of Glycated hemoglobin as well as adequate blood pressure and blood lipid level monitoring, self-efficacy, self-care behaviors as well as, requires dietary modifications, exercise, and administration of medication [11].

A Previous study conducted at Tikur Anbessa Specialized Hospital indicated a high \prevalence of poor glycemic control (68.3%). Other systematic reviews conducted in Ethiopia showed that the magnitude of good glycemic control was (34.4%and 33.2%) based on fasting plasma glucose and glycosylated hemoglobin measurements respectively [12, 13].

Even though a large number of studies were conducted across different African countries regarding the magnitude of poor glycemic control, its prevalence was high. This fact was revealed by a study conducted 71.9% in eastern Sudan, 64.9% in Nekemte Referral Hospital,74.0% in Cameroon, 61.3% in Zambia, 69.7% in Tanzania,75.2% in Senegal,79.2% in Uganda, and 62% in Nigeria. This high prevalence indicates broadly the fatal effect of high blood glucose suffering the life of the public across the continent [14–21].

Poorly controlled blood glucose causes a social nuisance, deterioration of healthy life, poor self-care practices, and lack of solidarity that increases their vulnerability to microvascular and macrovascular complications [22].

Studies conducted in various areas showed different factors causing poor glycemic control, which includes a late of beginning insulin, poor adherence to treatment, diet and exercise, younger age, hypertension, and non-adherence to diabetes self-management behaviors, no formal education, and being a farmer were independent determinants of poor glycemic control [23, 24].

Moreover, the study revealed that age, adherence to medication, duration of diabetes, physical activity, self-care behavior, and self-efficacy were predictors of glycemic control [25].

An inclusive self-management of diabetes includes self-monitoring of blood glucose; dietary restrictions, regular foot care, and ophthalmic examination have all been shown to reduce the incidence and progression of diabetes complications. This concluded that miss of implementation of one of the above treatment modalities devastates, mortality, and complication related to diabetes patients [26].

Several studies conducted regarding factors associated with glycemic control in Ethiopia were uni-facility based studies. The majority indicated that younger age, hypertension, poor adherence to the medication, no formal education, and farmer, taking a combination of insulin and oral medication, and smoking were predictors of poor glycemic control [27, 28].

However, key factors used for management modality that engages patients’ behavior change to develop prolonged interventions and close monitoring of glucose including self-efficacy, home blood glucose test, family history, and self-care behaviors were not studied well in patients with diabetes in Ethiopia. In particular, there was no study conducted regarding self-efficacy and special diet recommendations as predictors of poor glycemic control in the western part of Ethiopia. Thus, this study fills up these gaps and adds new predictors that are pivotal for delivering comprehensive care for patients with diabetes. Therefore, the study aimed to assess the magnitude and predictors of poor glycemic control among patients with diabetes on follow up at public hospitals of Western Ethiopia.

## Methods and materials

### Study setting and population

The study was conducted in multi-facility based public hospitals found in western Ethiopia from January 20-March 20, 2020. The institutional-based cross-sectional study design was employed. The public hospitals were selected randomly by lottery method from all public hospitals found in Western Ethiopia. The four selected hospitals were Wollega university referral hospital and Nekemte specialized hospitals which are found in Nekemte town at a distance of 331km from Addis Ababa. Nekemte town has a latitude and longitude of 9°5’N36°33’E and an elevation of 2,088 meters. Gimbi general hospital is found in Gimbi town West Wollega Zone. It has a latitude and longitude of 9°10’N35°50’E with an elevation between 1845 and 1930 meters above sea level. Shambu hospital is found in Shambu town Horo Guduru Wollega Zone. It has an elevation of 2,503 meters above sea level. The selected public hospitals were serving as primary services, general services, and specialized levels of services for more than a total population of 11 million for the Western part of Ethiopia.

### Study population and samples

Primarily, four public hospitals were selected randomly by a lottery method from all public hospitals found in Western Ethiopia. The sampling frame was taken from a medical record of the diabetes patients on follow- up receiving ant diabetic medication from the chronic outpatient department of respective hospitals. The study was conducted on a total of 1280 patients with diabetes on follow-up and receiving diabetic medications. A constant K value was calculated from a sampling frame. The value of K was obtained by dividing the total number of diabetes patients (1280) for the sample size (n = 423). The first record of a patient was selected by the lottery method and every Kth record (K = 3) was included. Finally, informed consent was provided for all selected participants. All diabetic patients attending selected hospitals were the source population and all the sampled patients with diabetic on follow- up receiving diabetic medication for at least six months and present during the data collection period were the study population. All diabetic patients on follow- up with measured A1C ≥7.0% (53mmol/mol)] were included. Participants taking anti-diabetic medication for less than six months and unconscious patients who had serious complications were unable to respond and were excluded from the study.

### Sample size determination and sampling techniques

The sample size of the study was calculated using the formula for estimation of a single population proportion with the assumptions of 95% Confidence Level (CL), marginal error (d) of 0.05. Despite the presence of a previous study, we used a 50% proportion to increase the sample size and representativeness of the sample that finally ensures the generalization and precision of the findings.

$$\text{Thus},\;\text{the}\;\text{Sample}\;\text{size}\;\text{was}\;\text{n}=\frac{\left(z1-1/2\right)2\times p\left(1-p\right)}{{\text{d}}^{2}}$$

$$\text{n}=\frac{{\left(1.96\right)}^{2}*0.5\left(1-0.50\right)}{\left(0.05\right)\;²}=385$$

Thus, by adding a non-response rate of 10% and using the correction formula; the final sample size was 423 people living with diabetes mellitus and treated with anti-hyperglycemic medication were enrolled in the study. Study participants were selected by using systematic random sampling techniques from each hospital.

### Data collection tool and procedures

Data collection tools consist of three-part questionnaires: The first part consists of demographic questions developed by investigators like sex, age, ethnicity, language, religion, educational status, occupation, marital status, and residence. The second part of the questionnaires includes clinical and behavior related variables such as smoking, duration of diseases, type of treatment, body mass index, weight, family history of DM, exercise, blood glucose test, and diabetes complications. Participants’ height and weight were measured as part of the physical examination. Height and weight were measured by trained and experienced BSc nurses recruited as data collectors working in different sites. Height and weight were measured by a Standiometer and weighing scale. All diabetes patients on follow- up attending respective hospitals had been included in the measurement to calculate body mass index. Body mass index was measured in terms of the patient’s weight in kilograms divided by the square of the patient’s height in meters (kg/m2). The interpretation of this result was based the World Health Organization criteria of body mass index classification and classified as <18.5kg/m2 (underweight), 18.5–24.9kg/m2 (normal weight), 25–29.9 kg/m2 (overweight) and that of ≥ 30kg/m2 (obesity).

The third part of the questionnaires measures self-care behaviors: The revised version of the Summary of Diabetes Self-Care Activities (SDSCA) questionnaire taken from the previous study originally developed by Schmitt et al was used to measure participants’ self-reported frequency of adhering to self-care behaviors [29]. The statistical method factor analysis was performed using the principal component method, Varimax rotation with Kaiser Normalization at Eigenvalue >1. The intraclass correlation coefficients of all questionnaires were checked. Some measurement tools with low interclass correlation coefficients below the acceptable standard (> 0.50) were reduced. Therefore, two items were removed and the remaining thirteen items with three dimensions were used to assess the participants’ frequency of engaging in diabetes self-care behaviors. The formulated three dimensions were glucose management (4 items), physical activity (4 items), and dietary control (5 items). Of the total thirteen items, six items were formulated positively and seven were negatively stated and reversed. Participants were asked to indicate the number of days they engaged in each of the self-care behaviors for the past 7 days. The greater the number of days reported for behavior the better the self-care. The scale was measured on a four-point Likert scale that starts from 0 = does not apply to me, 1 = applies to me to some degree, 2 = applies to me to a considerable degree, and 3 = applies to me very much, and the higher score indicated more effective self-care. The reliability of the summary of diabetes self-care activities was reported with Cronbach alpha = 0.86 which was acceptable.

The fourth part of the questionnaire was self-efficacy related questions collected via 8 modified questions from the diabetes mellitus self-efficacy scale (DMSES) adapted from the previous study originally developed by Wellston [30]. The responses were rated on a 5-point Likert scale: “1 = not confident, 2 = not very confident, 3 = confident half the time, 4 = usually confident, 5 = always confident. The lower score indicated poor/ fair self-efficacy and higher score high self-efficacy. The reliability of the diabetes mellitus self-efficacy scale was reported with Cronbach’s alpha = 0.880 which was acceptable.

Finally, The Glycated hemoglobin levels (A1C) of the participants were used as an indication of glycemic control. Glycosylated hemoglobin was obtained on the same day as the measure of glycemic control. A1C levels of participants were categorized according to American Diabetes Association into good glycemic control when A1C less than 7% (<53mmol/mol)] and poor Glycemic control when A1C greater than 7.0% (≥53mmol/mol)]. Three milliliters of venous blood samples of patients were taken and their Glycated hemoglobin was determined using the fast ion exchange resin separation method found on red blood cells for the previous three months. The sample was processed by a lab technician in the respective laboratory unit and finally, the result was recorded from the patient s’ lab result by data collectors. Thus, glycemic control was categorized according to American Diabetes Association as good glycemic control when glycosylated hemoglobin less than 7% (53mmol/mol)] and poor Glycemic control when glycosylated hemoglobin greater than 7.0% (53 mmol/mol)]. A Close-ended interviewer-administered structured questionnaire has interviewed the participants by trained data collectors. Six trained BSc nurses working in different areas of the study sites were recruited as data collectors and two of them were employed as supervisors for consecutive two months.

### Operational definition

#### Glycemic control

Is a medical term referring to levels of blood glucose in a person with diabetes mellitus which were categorized by American Diabetic Association (ADA) based on the value of glycosylated hemoglobin levels as follows [31, 32]:

#### Good glycemic control

The average glycosylated hemoglobin was less than 7% (<53 mmol/mol).

#### Poor glycemic control

The average glycosylated hemoglobin was greater/equal 7.0% (≥53 mmol/mol).

#### Self-efficacy

“The diabetes patients’ belief and judgment of their capability of carrying out diabetes self-management activities” (Bandura, 1994).

#### Poor self–efficacy

Those patients’ self-efficacy scored below the mean of self-efficacy scores.

#### High self–efficacy

Those patients self-efficacy scored above mean of self-efficacy scores.

#### Self-care behaviors

Defined as activities performed by diabetic patients including healthy eating plan, exercise, self-glucose monitoring, and diabetes medication and/or insulin intake.

#### Poor self-care behaviors

Those patients’ self- care behaviors scored less than 50th percentile of self- care behaviors scores.

#### Good self -care behaviors

Those patients’ self- care behaviors scored more than 50th percentile of self- care behaviors scores.

### Reliability of the instruments

The sampling adequacy was checked by the Kaiser Meyer Olkin test. For all scales, confirmatory factor analysis was performed to know the adequacy of all instruments (accepted standard >0.5). The intraclass correlation coefficient was used for testing the reliability of scales. Some items were reduced based on the value of the intraclass correlation coefficient using the principal component method, Varimax rotation with Kaiser Normalization at Eigenvalue >1.

A statistical method of factor analysis was performed in the summary of diabetes self-management tools with Kaiser-Meyer-Olkin Measure of Sampling Adequacy was 0.83(DF = 105, p = 00). The rotated component matrix of loading factors showed three dimensions (1, 2&3). The total variance explained for the first dimension was 23.44%, the 2nd dimension 39.28% and the third dimension was 57.69%. The overall Cronbach alpha of the tool was = 0.86. Similarly, confirmatory factor analysis was performed for the self-efficacy scale with Kaiser-Meyer-Olkin Measure of Sampling Adequacy was 0.86(DF = 28, p = 00). The rotated component matrix of loading factors resulted in two dimensions (1&2) with total variance explained for the 1st and 2nd dimensions (35.76% 68.76%) respectively. The overall Cronbach’s alpha = 0.88

### Ethical approval and consent to participate

The study was reviewed and approved by the Institutional Review Boards of Wollega university Ethical review board with a reference number of WU/133,224. The purpose of the study was explained to the medical director and staff of the hospital and permission was obtained. We conducted the study in accordance with the Declaration of Helsinki by including basic principles of ensuring the study subject’s privacy, risk, and benefit, conducted by trained professionals, written informed consent obtained, and even we allowed the right to withdraw if the study participants requested. The consent was obtained from the participants themselves. Moreover, the confidentiality of the information was assured.

### Data quality control

All questionnaires were adopted in the English language translated into the local language Afan Oromo and then re-translated back into English by experts. A pretest was conducted on 5% of the sample size at Gimbi Adventist hospital that was outside the actual study setting before data collection. The training was given one day for both data collectors and supervisors. Data were cleaned, coded, and checked for consistency and completeness. A consistency was checked by a double-entry method to improve the quality of the data, and inconsistent entries and responses were crosschecked.

### Data processing and analysis

Data were cleaned, edited, coded, and entered into Epi data version 3.1 and was exported to SPSS windows version 24 for analysis. Descriptive statistics including, percentage, ratios, frequency distribution, mean and standard deviation, and pie chart was used to describe the data. Normalization of the data was checked using histograms, normal Q-Q, Scatter plot, Hosmer and Lemeshow test was used to know the homogeneity of variables. Multicollinearity was checked using tolerance and the Variance Inflation Factor (VIF). All variables significant at p-value <0.25 in the bivariable were entered in multivariate logistic regression analysis. Backward stepwise goodness of fit was used to ascertain the suitable variables in multivariable logistic regression analysis. Finally, multivariate logistic regression analysis with AORs, CI at 95%, and the significance level was set at p <0.05.

## Results and discussions

### Socio-demographic characteristics of participants

Four hundred twenty-three participants participated giving a response rate of 94.10%. More than half 210(52.76%) of the participants were male with a median age of 45(±15.88SD). The majority of the participants 168(42.21%) were aged above 50 years followed by 40–49 96(24.12%) years. Concerning their ethnicity, the majority of the participants 355(89.20%) were Oromo with a language speaker 327(82.16%) Afan Oromo. Nearly half 189(47.49%) of the respondents were protestant followers followed by 134(33.67%) was an orthodox believer. Concerning marital status, about 308(77.39%) were married (Table 1).

**Table 1:** Distribution of socio-demographic characteristics of diabetes patients attending public hospitals of western Ethiopia (N = 398).

Variables	Category	Number (%)
Sex	Male	210(52.76)
	Female	188(47.24)
Age	20–29	66(16.58)
	30–39	68(17.09)
	40–49	96(24.12)
	≥50	168(42.21)
Ethnicity	Oromo	355(89.20)
	Amhara	38(9.55)
	Others©	5(1.26)
Mother tongue language	Afan Oromo	327(82.16)
	Amharic	62(15.58)
	Others	9(2.26)
Religion	Orthodox	134(33.67)
	Muslim	59(14.82)
	Protestant	189(47.49)
	Others®	16(4.02)
Educational status	No formal education	97(24.37)
	Elementary	96(24.12)
	High school	96(24.12)
	College/university	109(27.39)
Marital status	Married	308(77.39)
	Unmarried	90(22.61)
Occupation	Daily laborer	48(12.06)
	Merchant	87(21.86)
	Farmer	94(23.62)
	Employee	169(42.46)
Residence	Urban	246(61.81)
	Rural	152(38.19)

Others© = (Tigre, Silte, Gumuz), Others® = (Wakefata).

### Clinical characteristics of participants

The study results showed most of the participants 222(55.78%) were smokers. The majority of 303(76.13%) of the respondents had no family history of diabetes. More than half 214(53.77%) of patients with diabetes were suffering for 5-7years. Concerning diabetes complications, about 271(68.09%) of participants had no diabetes-related complications. Approximately three-fourth 295(74.12%) of the respondents did physical activity per week and nearly half 203(51.01%) of them test their blood glucose at home. About 279(70.10%) of the participants had no special diet. The majority of the patients 285(71.61%) had been taking a combination of oral hypoglycemic agents and insulin (Table 2).

**Table 2:** Proportion of clinical characteristics of diabetes patients attending public hospitals of western Ethiopia (N = 398).

Variables	Category	Number (%)
Smoking status	Nonsmokers	176(44.22)
	Smokers	222(55.78)
Duration of diabetes	1–4 years	79(19.85)
	5-7years	214(53.77)
	≥8 years	105(26.38)
Types of treatment	Non pharmacological	20(5.03)
	Insulin	93(23.37)
	OHA+ insulin	285(71.61)
Body mass index (Kg/m^2^)	<18.5	157(39.45)
	18.5–24.9	103(25.88)
	25–29.9	101(25.38)
	≥30	37(9.30)
Weight change (kg)	Weight gain	192(48.24)
	Weight loss	206(51.76)
Family history	Yes	95(23.87)
	No	303(76.13)
Special diet	Yes	119(29.90)
	No	279(70.10
Exercises	Yes	295(74.12)
	No	103(25.88)
Blood glucose test	Yes	203(51.01)
	No	195(48.99)
Diabetes complication	Yes	127(31.91)
	No	271(68.09)

### Prevalence of self-care behavior among patients with diabetes attending public hospitals of western Ethiopia

**Fig 1:**
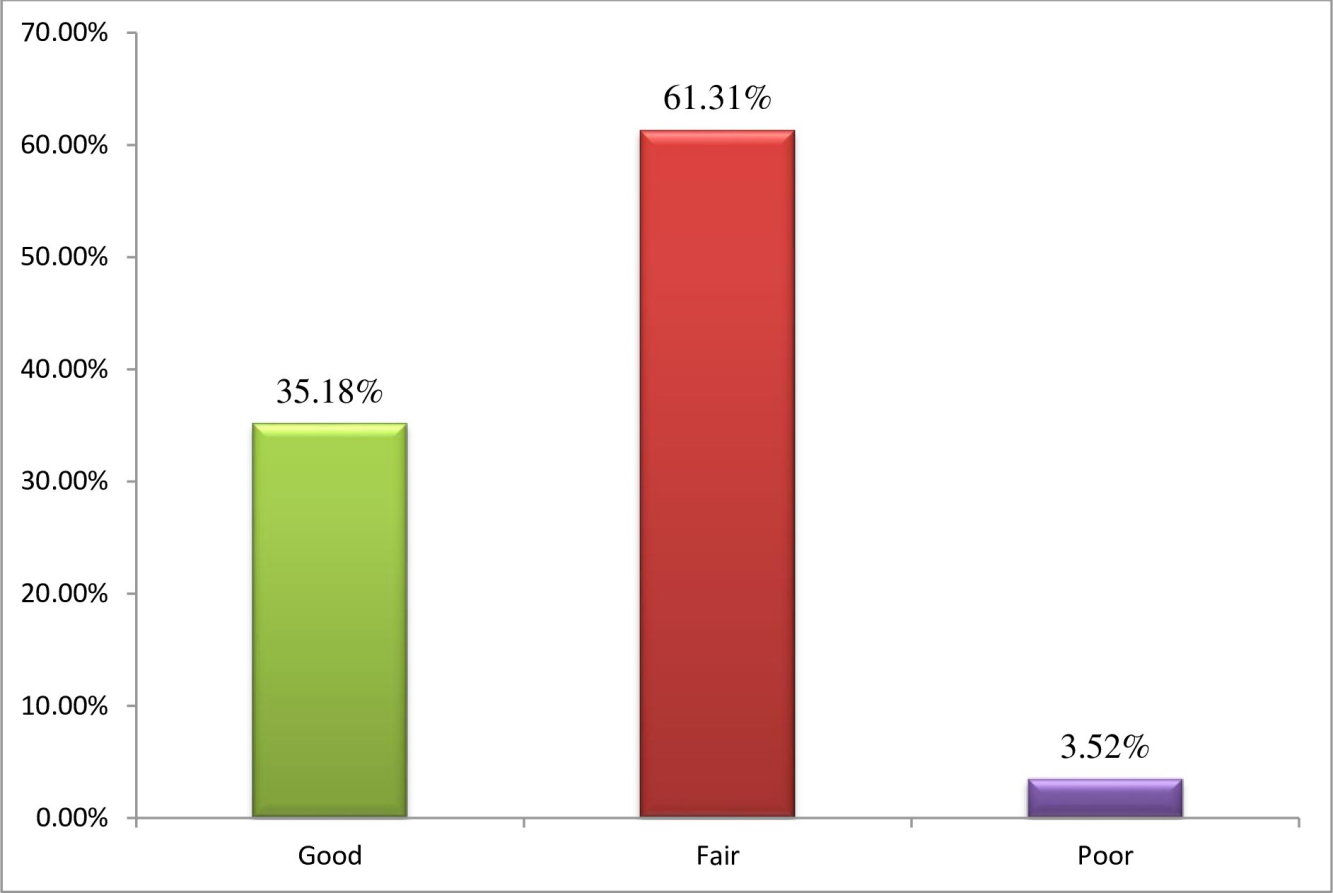
Prevalence of self-care behavior among patients with diabetes attending public hospitals of western Ethiopia.

The overall level of practice of self-care behavior was classified as poor, fair, and good self-care behaviors using less than 25th, 25th-75th, and more than the 75th percentile of their possible scores. Thus, of 398 respondents the prevalence of good self-care behavior 140 (35.18%), fair self-care behavior 244 (61.31%), and about 14(3.52%) were poor self-care behavior (Fig 1).

### The magnitude of glycemic control among patients with diabetes attending public hospitals of western Ethiopia

**Fig 2:**
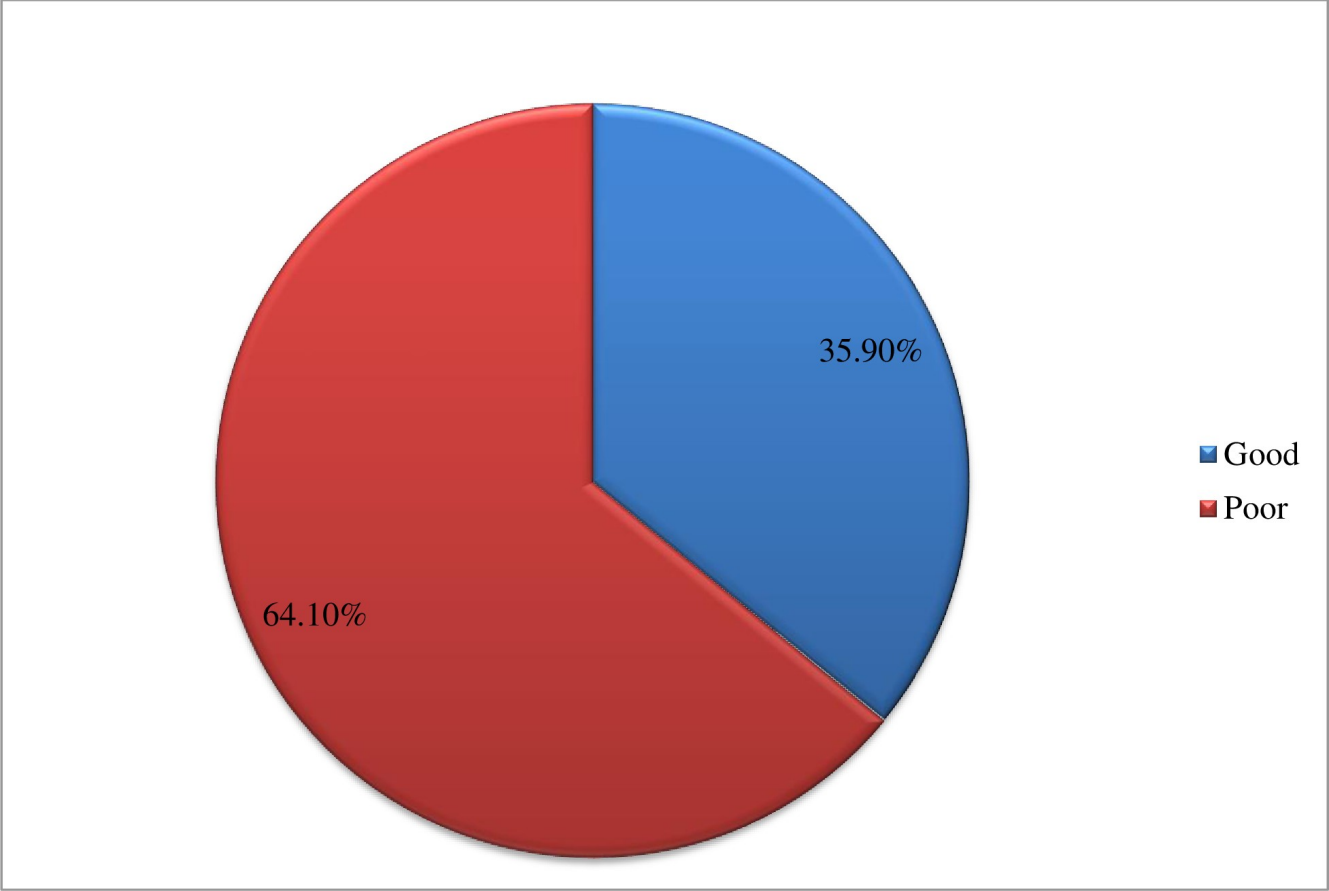
The magnitude of glycemic control among patients with diabetes attending public hospitals of western Ethiopia.

Glycemic control was classified as good and poor glycemic control based on the value of glycosylated hemoglobin levels developed by the American Diabetic Association. Therefore, the prevalence of poor glycemic control was 64.1% (HbA1c ≥7%) and the remaining 35.9% was good glycemic control (HbA1c ≤7%) (Fig 2).

### Bivariable logistic regression analysis of factors associated with glycemic control

In logistic regression, poor self-efficacy, poor self-care behavior, being female, presence of diabetes complications, absence of blood glucose, weight gain, duration of diabetes >8years, being smoker, were variables associated with poor glycemic control at P <0.25 (Table 3).

**Table 3:** Bivariable logistic registration analysis of factors associated with poor glycemic control among diabetes patients attending public hospitals of western Ethiopia, 2020.

Variables	Category	Glycemic control		COR (95% CI)	P- value
		Poor	Good		
Sex	Male	123(58.6%)	87(41.4%)	1	
	Female	132(70.2%)	56(29.8%)	1.667(1.100,2.527)	0.016
Smoking status	Non smoker	86(48.9%)	90(51.1%)	1	
	Smoker	169(76.1%)	53(23.9%)	3.337(2.177,5.116)	0.001
Weight change (Kg)	Weight gain	129(67.2%)	63 (32.8%)	1.300 (0.862,1.962)	0.211
	Weight loss	126 (61.2%)	80 (38.8%)	1	
Body mass index (Kg/m^2^)	<18.5	31(83.8%)	6(16.2%)	1	
	18.5–24.9	64(62.1%)	39(37.9%)	0.318(0.122,0.830)	0.019
	25–29.9	95(60.5%)	62(39.5%)	0.297(0.117,0.752)	0.01
	≥30	65(64.4%)	36(35.6%)	0.349(0.133,0.917)	0.033
Diabetes complication	No	152(56.1%)	119(43.9%)	1	
	Yes	103(81.1%)	24(18.9%)	3.306(1.995,5.478)	0.001
Blood glucose test	No	139(71.3%)	56(28.7%)	1.862(1.227,2.824)	0.003
	Yes	116(57.1%)	87(42.9%)	1	
Duration of diabetes	1–4 years	124(57.9%)	90(42.1%)	1	
	5–7 years	45(57.0%)	34(43.0%)	0.961(0.570,1.618)	0.88
	>8years	86(81.9%)	19(18.1%)	3.285(1.865,5.787)	0.001
Types of DM treatment	Non pharmacological	15(75.0%)	5(25.0%)	2.00(0.707,5.656)	0.191
	Insulin	69(74.2%)	24(25.8%)	1.917(1.138,3.229)	0.014
	OHA+ Insulin	171(60.0%)	114(40.0%)	1	
Self-care behavior	Good	153(59.3%)	105(40.7%)	1	
	Poor/Fair	102(72.9%)	38(27.1%)	1.842(1.510,4.027)	0.007
Self-efficacy	Poor	93(77.5%)	27(22.5%)	2.466(1.510,4.027)	0.00
	High	162(58.3%)	116(41.7%)	1	

Significance at P-value < = 0.25, Dependent variable = Glycemic control.

### Multivariable logistic regression analysis of factors associated with poor glycemic control

All variables significant at the bivariate level were entered into multivariable logistic regression analysis. In multivariable logistic regression analysis, seven variables were significantly associated with poor glycemic control including didn’t test blood glucose, presence of diabetes complications, being female, Duration of diabetes> 8 years, low self-efficacy, poor self-care behaviors, Body mass index >30Kg/m2. Being females were two times more likely to have poor glycemic control compared to males (AOR = 1.684, 95%CI = 1.066, 2.662). Concerning the duration of diabetes patients who had > 8 years were three times more likely to have poor glycemic control compared to 1–4 years (AOR = 2.552, 95%CI = 1.397,4.665). Participants who had diabetes complications were three times more likely to have poor glycemic control compared to didn’t have diabetes complications (AOR = 2.806, 95%CI = 1.594, 4.941). Participants who didn’t test blood glucose at home were two times more likely to have poor glycemic control compared to those who tested blood glucose at home (AOR = 1.720, 95%CI = 1.078,2.743). Participants who had poor self-care behavior were two times more likely to have poor glycemic control compared to good self-care behaviors (AOR = 1.787, 95%CI = 1.083, 2.959). Participants who had poor self-efficacy were two times more likely to have poor glycemic control compared to good self-efficacy (AOR = 1.934, 95%CI = 1.078,3.469) (Table 4).

**Table 4:** Multivariable logistic registration analysis of factors associated with glycemic control among diabetes patients attending public hospitals of western Ethiopia, 2020.

Variables	Category	Glycemic control		COR (95% CI)	P- value	AOR (95% CI)	P- value
		Poor	Good				
Sex	Male	123(58.6%)	87(41.4%)	1		1	
	Female	132(70.2%)	56(29.8%)	1.667(1.100,2.527)	0.016*	1.684(1.066,2.662)	0.026**
Smoking status	Non smoker	86(48.9%)	90(51.1%)	1			
	Smoker	169(76.1%)	53(23.9%)	3.337(2.177,5.116)	0.001*		
Weight change (Kg)	Weight gain	129(67.2%)	63 (32.8%)	1.300 (0.862,1.962)	0.211		
	Weight loss	126 (61.2%)	80 (38.8%)	1			
Body mass index (Kg/m^2^)	<18.5	31(83.8%)	6(16.2%)	1		1	
	18.5–24.9	64(62.1%)	39(37.9%)	0.318(0.122,0.830)	0.019	0.278(0.101,0.768)	0.013**
	25–29.9	95(60.5%)	62(39.5%)	0.297(0.117,0.752)	0.01	0.302(0.113,0.804)	0.017**
	≥30	65(64.4%)	36(35.6%)	0.349 (0.133,0.917)	0.033	0.325(0.118,0.899)	0.030**
Diabetes complication	No	152(56.1%)	119(43.9%)	1		1	
	Yes	103(81.1%)	24(18.9%)	3.306(1.995,5.478)	0.00*	2.806(1.594,4.941)	0.001**
Blood glucose test	No	139(71.3%)	56(28.7%)	1.862(1.227,2.824)	0.003*	1.720(1.078,2.743)	0.023**
	Yes	116(57.1%)	87(42.9%)	1		1	
Duration of diabetes	1–4 years	124(57.9%)	90(42.1%)	1		1	
	5–7 years	45(57.0%)	34(43.0%)	0.961(0.570,1.618)	0.88	0.673(0.380,1.193)	0.175
	>8years	86(81.9%)	19(18.1%)	3.285(1.865,5.787)	0.00*	2.552(1.397,4.665)	0.002**
Types of DM treatment	Non pharmacologic	15(75.0%)	5(25.0%)	2.00(0.707,5.656)	0.191		
	Insulin	69(74.2%)	24(25.8%)	1.917(1.138,3.229)	0.014*		
	OHA+Insulin	171(60.0%)	114(40.0%)	1			
Self-care behavior	Poor/Fair	153(59.3%)	105(40.7%)	1.842(1.510,4.027)	0.007*	1.787(1.083,2.959)	0.023**
	Good	102(72.9%)	38(27.1%)	1		1	
Self-efficacy	Poor	93(77.5%)	27(22.5%)	2.466(1.510,4.027)	0.00	1.934(1.078,3.469)	0.027**
	High	162(58.3%)	116(41.7%)	1		1	

* = Significance at P-value< = 0.25,

** = Significance at P-value < = 0.05, Dependent variable = Glycemic control.

## Discussions

The implementations of self-monitoring blood glucose strategies have been significantly controlled blood glucose levels in patients with diabetes mellitus. However, the strategy is being challenged by poor adherence to self-care practices and the presence of secondary complications that results from high blood sugar levels. Therefore, routine monitoring of blood glucose levels and identifying its underlying predictors associated with poor glycemic control are vital to prevent the development of secondary complications related to poor glycemic control. The overall prevalence of poor glycemic control in this study was 64.1% attaining A1C more than 7% (>53mmol/mol) which was comparable with the result of a study conducted in Nekemte Referral Hospital (64.9%), and Limu Genet hospital, southwest Ethiopia revealed (63.8%) of participants had poor glycemic control. However, it was relatively higher than the result of the study done in Nigeria (59.4%) and China (47.3%) of the study participants achieving A1C greater than 7%. On the contrary, this finding was lower than the result of the studies conducted in Kenya (81.9%) and Thailand (72.1%) of the study participants had poor glycemic control. These differences might be due to variations in nutritional habits, living standards, and knowledge on prevention and treatment strategies across the countries [15, 33–37].

The intervention techniques to control blood glucose through practicing self-monitoring blood sugar in terms of nutrition, exercise, and closely monitoring blood glucose are the cornerstone. In this study adhering to self-care behavior was an independent factor associated with glycemic control bearing in mind the joint effect of other variables. Patients adhering to poor self-care behavior were more likely to increase the odds of poor glycemic control two times compared to good self-care behaviors. This finding was similar to a study conducted in Jordan that revealed an increased score of barriers to adherence self-care behaviors were significantly associated with increased odds of being poorly controlled blood sugars. However, it was contradictory with the study conducted in Ghana which explained that diabetes self-management has a negative correlation with poor glycemic control. This disparity might denote the presence of other confounding factors related to poor glycemic control [38, 39].

The current study also revealed that increased duration of diabetes mellitus was more likely associated with poor glycemic control compared to a short duration of diabetes less than 4 years. This finding was comparable to a study conducted in China which revealed patients who had >4years were two times more likely to had poor glycemic control compared to a duration less than< 1 year which was consistent with a study conducted in Indonesia showed that increasing duration of diabetes > 5 years significantly influence poor glycemic control with increased odds ratio scores. These similarities might signify increased duration of the disease process, continuously decreased insulin production, and encouraged incidence of diabetes complications that finally increased blood glucose level [40, 41].

The finding of this study also showed that lack of blood glucose test at home was two times more likely associated with poor glycemic control compared to patients who test blood glucose at home. This finding was comparable to the study done at Tikur Anbessa hospital which showed those who have a glucometer at home were eight times more likely to have good glycemic control compared with those who didn’t have it. This resemblance justifies the fact that close monitoring of blood glucose at home was basic strategies to reduce blood glucose [42].

In this current study respondents who had diabetes complications were three times more likely to have poor glycemic control compared to those who did not have diabetes complications which were similar to a study conducted in the Illubabor zone Oromia region, Ethiopia showed patients who did not have comorbidities illness were three times more likely to have good glycemic control compared to those who had diabetes complications. The possible similarity might be because the presence of comorbid illness aggravates disease processes and reduces their quality of life [43].

According to this study, the sex of the patient is one of the significant predictors of poor glycemic controls. The odds of being female were two times more likely to have poor glycemic control compared to males which are similar to a study conducted in Nigeria which showed that a larger proportion of female patients had uncontrolled blood glucose which was analogous with the study conducted in Kenya which enlighten poor glycemic control was significantly higher in females than in males. The possible similarities might be related to the higher natural deposit of fat contents in females which facilitate insulin resistance than males [44, 45].

Moreover, this study also showed that poor self-efficacy was more likely associated with poor glycemic control compared with good self-efficacy which was similar to a study done in Sudan which showed that patients who had high self-efficacy were two times more likely controlled blood glucose compared to with low self-efficacy. This similarity justifies the fact that diabetes patients’ confidently engaging in self-monitoring their blood glucose was fundamental to control diabetes [46].

## Conclusion

The proportion of poor glycemic control was high which was nearly comparable to that reported from many countries. This could be due to factors that were significantly associated with poor glycemic control like lack of home blood glucose test, increased duration of diabetes, presence of diabetes complications, poor self-efficacy, and poor self-care behaviors were significant independent predictors of poor glycemic control. Thus, we recommend patients with diabetes and health care providers enhancing self-monitoring practices, and preventing potential complications should be a priority concern to improve blood glucose levels. enhancing self-monitoring practices, preventing potential complications should be a priority concern to improve blood glucose levels. Further studies are also recommended to explore important factors that were not identified by the current study.

### Limitations of the study

The research design cannot confirm causality.

There were social desirability biases expressed by the respondents.

### Study implications

The findings of the study found important parameters related to poor glycemic control including low self-efficacy, absence of blood glucose test, presence of diabetes complication, poor self-care behavior, being obese. These parameters were important implications of this study for integrating the national health policy-making for practicing self-monitoring blood glucose and reducing secondary complications to blood sugar.

## Supporting information

S1 Questionnaries English version questionnaires. (DOCX)

S1 Dataset (SAV)

## Abbreviations

ADA: American Diabetes Association
AOR: Adjusted Odds Ratio
BMI: Body Mass Index
DM: Diabetes Mellitus
DMSES: Diabetes Mellitus Self-efficacy Scale
DSMQ: diabetes self-management questionnaire
HIV: Human Immunodeficiency Virus
SD: Standard Deviation
SMBG: self-monitoring of blood glucose
VIF: Variance Inflation Factor
