# Supplementary material for: Magnitude and predictors of poor glycemic control among patients with diabetes attending public hospitals of Western Ethiopia
Source: PLoS One. 2021 Feb 25;16(2):e0247634. doi: 10.1371/journal.pone.0247634 (PMC7906479; doi:10.1371/journal.pone.0247634)
Supplement: S1 Questionnaries — (DOCX) [file pone.0247634.s001.docx]

# English Version Questionnaires

## Part I: Personal Information

001. Age in year: _____________002. Height (cm):__________ 003. Weight (kg): ____________

004. Sex: 1. Male 2. Female

005. Marital Status: 1. Single 2. Married 3. Divorced 4.Widowed

006. Religion

1. Orthodox
2. Muslim
3. Protestant
4. Catholic
5. Other(Specified) _______

007**.** Educational status

1. No formal education
2. Elementary
3. High school
4. College/University

008. Residence 1. Urba. 2. Rural

009. Ethnicity 1. Oromo 2. Amhara 3.Tigre 4. Others (specify)

010. Language (mother tongue language )

1. Afan oromo 2. Amharic 3.tigrigna 4.Walaytigna 5. Others (specify)

011. Occupation:

1. Daily laborer
2. Merchant
3. Farmer
4. Employee
5. Others (specify )

**Part II: Clinical characteristics information**

012. Smoking Status:

1. Nonsmoker
2. Current Smoker
3. Former smokers and quit since ___(state the period)

013. Duration of diabetes since

1. = 1 year
2. 2-4 years
3. 5-7 years
4. >8 years

014. Family History of Diabetes:

1. First-degree relatives (parents - Siblings - children)
2. Second-degree relatives (grandparents - aunts / uncles & their children)
3. No family History
4. Don’t Know

015. Specify your Diabetes Treatment

1. Diet only
2. Medication/Insulin Only
3. Medication/Insulin + Diet
4. Diet + Exercise

016. How many times do you take your medicine/insulin per day?

1. Never
2. Once a day
3. Two times a day
4. Three or more a day

017. With which meals are you taking your medication/insulin?

1. Breakfast 2. Lunch 3.Dinner

018. Are you on special diet?

1. No
2. Yes, given by physician
3. Yes, given by dietitian
4. Yes, self-arranged

019. How often do you exercise or do physical activity per week?

1. None
2. 1-2 times week
3. 3-4 times week
4. 5-6 times week
5. Daily

020. How frequently do you test your blood glucose at home?

1. None
2. 1-2 per week
3. 3-4 per week
4. 5-7 per week
5. Only when feeling diabetes symptoms

021. Do you have any diabetes-related complications? (Select all that apply)

1. None
2. Eye Disease
3. Heart Disease
4. Foot Gangrene/Amputation
5. Nerve Disease
6. Diabetic Coma
7. Kidney Disease
8. Others specify __________

022. How would you describe your appetite?

1. Very Good
2. Good
3. Fair
4. Poor

023. How many meals and snacks do you eat during the day?

1. 1-2 meals
2. 3 meals
3. 4-5 meals
4. 6 meals or more

024. What other diet restrictions have you been told by your dietitian or physician to follow?

1. None
2. Low calorie
3. Low cholesterol
4. Low fat
5. Low salt/sodium
6. Low protein
7. High fiber
8. Other specify______

025. Has your weight changed in the past year?

1. No change 2. Gained weight ______kg 3. Lost weight______ kg

026. What is your blood glucose measurement____________(FBG/RBG/HgA1C/OGTT)?

## Part III- Summary of Diabetes Self-Care Activities Questionnaires (DSMQ)

**Instruction**: The following statements describe self-care activities related to your diabetes. Thinking about your self-care over the past 7 days, please specify the extent to which each statement applies to you described in terms of 0=Does not apply to me, 1=Applies to me some degree, 2=Applies to me a considerable degree and 3= Applies to very much

| S/no | statements | Level of performance | | | |
| --- | --- | --- | --- | --- | --- |
|  |  | Applies to me very much | Applies to me to a consider-able degree | Applies to me to some degree | Does not apply to me |
|  | **‘Glucose Management’ items** |  |  |  |  |
| 1. 1 | I check my blood sugar levels with care and attention. |  |  |  |  |
| 1. 22 | I take my diabetes medication (e. g. insulin, tablets) as prescribed. |  |  |  |  |
|  | I record my blood sugar levels regularly (or analyses the value chart with my blood glucose meter). |  |  |  |  |
|  | I do not check my blood sugar levels frequently enough as would be required for achieving good blood glucose control. |  |  |  |  |
|  | ‘**Dietary Control’ items** |  |  |  |  |
|  | I strictly follow the dietary recommendations given by my doctor or diabetes specialist. |  |  |  |  |
|  | Sometimes I have real ‘food binges’ (not triggered by hypoglycemia). |  |  |  |  |
|  | Occasionally I eat lots of sweets or other foods rich in carbohydrates |  |  |  |  |
|  | The food I choose to eat makes it easy to achieve optimal blood sugar levels. |  |  |  |  |
|  | I keep all doctors’ appointments recommended for my diabetes treatment. |  |  |  |  |
|  | **Physical Activity’ items** |  |  |  |  |
|  | I do regular physical activity to achieve optimal blood sugar levels. |  |  |  |  |
|  | I tend to avoid diabetes-related doctors’ appointments. |  |  |  |  |
|  | I avoid physical activity, although it would improve my diabetes. | ‘ |  |  |  |
|  | I tend to skip planned physical activity. |  |  |  |  |

**Part IV- Diabetes Mellitus Self-Efficacy Scale Questionnaires**

**Instruction**: The following statements describe self-efficacy related to your diabetes. The responses were rated on a 5-point Likert scale: “1=not confident, 2=not very confident, 3=confident half the time, 4=usually confident, 5=always confident.

|  | **statements** | **level of confidence** | | | | |
| --- | --- | --- | --- | --- | --- | --- |
|  |  | 1=not confident | 2=not very confident | 3=confident half the time | 4=usually confident | 5=always confident |
|  | How confident do you feel that you can eat your meals every 4–5 h every day, including breakfast every day? |  |  |  |  |  |
|  | How confident do you feel that you can follow your diet when you have to prepare or share food with other people who do not have diabetes? |  |  |  |  |  |
|  | How confident do you feel that you can choose the appropriate foods to eat when you are hungry (for example, snacks)? |  |  |  |  |  |
|  | . How confident do you feel that you can exercise 15–30 min, 4 to 5 times a week? |  |  |  |  |  |
|  | How confident do you feel that you can do something to prevent your blood sugar level from dropping when you exercise? |  |  |  |  |  |
|  | How confident do you feel that you know what to do when your blood sugar level goes higher or lower than it should be? |  |  |  |  |  |
|  | How confident do you feel that you can judge when the changes in your illness mean you should visit the doctor? |  |  |  |  |  |
|  | How confident do you feel that you can control your diabetes so that it does not interfere with the things you want to do? |  |  |  |  |  |
